# Weight Change and the Risk of Micro and Macro Vascular Complications of Diabetes: A Systematic Review

**DOI:** 10.21315/mjms2024.31.3.2

**Published:** 2024-06-27

**Authors:** Seyyed Kiarash Sadat Rafiei, Fardad Fateh, Mahla Arab, Mohammad Espanlo, Saba Dahaghin, Helia Karami Gilavand, Mehregan Shahrokhi, Mohammad Sadegh Fallahi, Zahra Zardast, Arina Ansari, Seyyed Alireza Seifhashemi, Ali Kheirandish, Gisou Erabi, Fatemeh Ahmadi Hajikolaei, Mahdi Nakhaee, Niloofar Deravi

**Affiliations:** 1 Student Research Committee, School of Medicine, Shahid Beheshti University of Medical Sciences, Tehran, Iran; 2 School of Medicine, Tehran University of Medical Sciences, Tehran, Iran; 3 School of Medicine, Shiraz University of Medical Sciences, Shiraz, Iran; 4 Student Research Committee, School of Medicine, North Khorasan University of Medical Sciences, Bojnurd, Iran; 5 Department of Pharmacology, School of Medicine, Iran University of Medical Sciences, Hemmat Highway, Tehran, Iran; 6 Student Research Committee, Urmia University of Medical Sciences, Urmia, Iran; 7 School of Medicine, Babol University of Medical Sciences, Babol, Iran; 8 Department of Laboratory Medicine, Faculty of Paramedical Sciences, Tehran Medical Sciences, Islamic Azad University, Tehran, Iran

**Keywords:** weight change, diabetes, cardiovascular, microvascular, body weight

## Abstract

Type 2 diabetes mellitus (T2DM) is a metabolic disease that can be a significant cause of cardiovascular disease (CVD), leading to macrovascular and microvascular diseases. Many researchers around the world have investigated the effects of weight change on micro and macro CVD in patients with T2DM. This study aimed to investigate the effect of weight change (weight gain and loss) on microvascular and macrovascular complications in patients with T2DM. We searched PubMed, Scopus and Google Scholar from the database until January 2023. We screened the title, abstract, and full text of articles, and after quality assessment, we extracted data from interrelated ones into this systematic review. Reviewing the results of 11 cohort studies with 219,839 individuals (T2DM patients) showed that weight loss caused an increase in the mortality rate in diabetic patients, while weight gain after diabetes diagnosis increased the risk of CVD, chronic kidney disease (CKD), microvascular disease, stroke and mortality. It should be noted that severe body weight variability increases the mortality rate and the risk of microvascular disease. Unlike other studies, one study showed that more than 5% weight gain positively affected CVD and coronary heart disease in T2DM patients. Generally, weight change in patients with T2DM is an essential sign of cardiovascular complications. According to our findings, the risk of cardiovascular complications in patients with weight loss is seen to be higher than in patients with weight gain. In regular patients with body mass index (BMI), stable weight in a healthy range is reported to decrease the risk of CVD.

## Introduction

Diabetes mellitus is a metabolic disease in which resistant hyperglycaemia occurs. Due to the chronic nature of this disease, elevated blood glucose levels can cause long-term macrovascular problems (for example, coronary artery disease or peripheral vascular disease) and microvascular diseases (retinopathy, nephropathy and neuropathy) (1). These complications could impair the normal function of several vital organs, thus increasing diabetes-related mortality among affected people. Rigorous adherence to glucose control and risk-modifying plans is necessary to avoid and reverse unfavourable outcomes. A growing body of evidence suggests an underlying inflammatory process that explains the pathogenesis of diabetes-related complications. Diabetes mellitus provides an environment where the local expression and release of cytokines, chemokines and growth factors increase, leading to tissue damage, including kidneys, arteries and the nervous system (2, 3).

Interestingly, obesity, a widespread phenomenon among the population with type 2 diabetes mellitus (T2DM), triggers similar inflammatory responses (4, 5). Therefore, the potential role of weight gain as an amplifier of inflammation and weight loss as a protective factor should be used. In this regard, multiple studies have sought to explore this relationship. Polemiti et al. (6) reported a positive link between body mass index (BMI) modulation and vascular complications in diabetic individuals. In this study, a decrease in BMI early after diabetes diagnosis contributed to a reduction in micro-vascular complications, nephropathy and neuropathy. However, this conclusion is disputed among similar studies, which many supports (7, 8) and some reject this linear relationship (9, 10). Therefore, this study aims to investigate the effect of weight change (both weight gain and loss) on micro- and macro-vascular complications in patients with T2DM and to address the discrepancy between existing research and studies.

## Method

The current systematic review protocol was registered on open scientific framework (OSF) (https://osf.io/ygtw3). We adhered to the Preferred Reporting Items for Systematic Reviews and Meta-Analyses (PRISMA) statement as a guide for conducting this review.

### Search Strategy

We thoroughly searched PubMed, Scopus and Google Scholar databases up to January 2023. We used a set of keywords pertinent to the change in weight and BMI, microvascular diseases (retinopathy, neuropathy and nephropathy) and macrovascular diseases (cardiovascular disease [CVD]) and study design. Table 1 summarises the search strategy for this study. The search included only articles in English.

### Eligibility and Study Selection

Three authors (SKSR, ME and MA) separately reviewed the title and abstract of all articles and excluded articles that did not meet the following criteria: i) studies designed as prospective cohorts or case-control; ii) studies only with participants 18 years old or older; iii) reporting unintentional weight gain during adulthood or prior to baseline evaluation in at least three quantitative categories (one category as stable weight/reference and two categories of weight gain); iv) reporting details of microvascular (retinopathy, neuropathy and nephropathy) and macrovascular (CVD) diseases, including incidence, mortality, heart failure, chronic heart disease (CHD), myocardial infraction (MI), stroke, as adverse outcomes; v) providing 95% confidence intervals (95% CI) for controlled risk estimates (relative risk [RR], risk ratio (95% CI) and vi) reporting the number of cases with T2DM/non-cases or person-years in each category of weight increase. Furthermore, studies that included only self-reporting weight gain were also selected. Review articles, editorials, commentaries and randomised control trial articles were excluded.

### Data Extraction and Assessment for Study Quality

Six others independently retrieved the following data from relevant studies: first author’s name, publication year, study name, country, age range and/or mean age (years old), number of participants (patients with T2DM), duration of follow-up, gender of participants (patients with T2DM) (%), duration of weight gain assessment, outcome and confounding factors included in the multivariate analysis. Adjusted RRs provided by multivariate analysis with the most significant confounders were considered from each study. The Newcastle-Ottawa scale was used to assess the quality of the included research. In our systematic search, we found three abstracts with no full text. We contacted the authors for additional information. One author provided us with the full text of one of them, but the study was not eligible and was not included in the systematic review. Discrepancies were resolved by discussion with the respective author’s supervision (SKSR).

## Results

**Figure 1:**
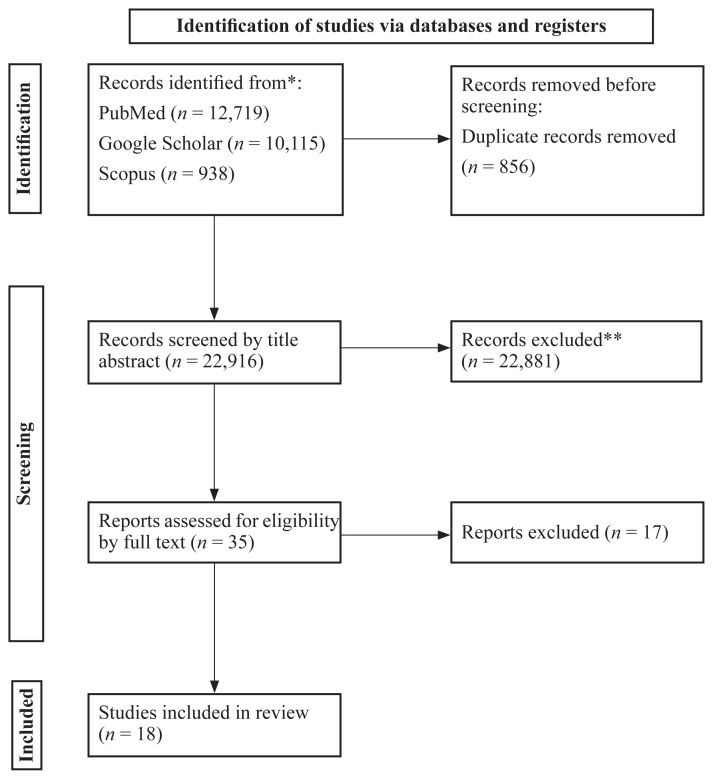
PRISMA 2020 flow diagram Notes: *Consider, if feasible to do so, reporting the number of records identified from each database or register searched (rather than the total number across all databases/registers); **If automation tools were used, indicate how many records were excluded by a human and how many were excluded by automation tools

We found 12,719 articles in PubMed, 938 articles in Scopus and 10,115 articles in Google Scholar (Figure 1). After selecting by title and abstract, and removing duplicates (856), 35 studies remained. After the full text and risk of bias assessment, 17 articles were excluded. Eleven cohort studies with 219,839 individuals (T2DM patients) were included to evaluate the effect of weight change and the risk of microvascular and macrovascular complications (6, 10–19). The average duration of follow-up was approximately 12.14 years, ranging from 3 to 38. The mean age of the individuals was 60.97 years old. Six studies (10–12, 14, 19, 20) defined the categories of weight change categories based on the percentage of individual weight change. Two studies (13, 17) defined mentioned categories based on the amount of weight change (5 kg to more than 10 kg for weight loss and 0 kg to more than 40 kg for weight gain). Three studies (6, 16, 18) defined the changes based on the BMI (percent or absolute change). Among these 11 studies, two evaluated weight change by self-reporting approach (6, 13) and eight (10–12, 14–16, 18, 19) by measurement. Only one study (17) used both methods. The normalised quality score of nine studies was 8 or greater than 8 (6, 11–15, 17–19) and only two were 7 to 8 (10, 16). Most of the articles used age, duration of diabetes, BMI, weight/weight change, heart disease, sex and smoking as adjusted variables. Reviewing the results of studies showed that weight loss caused an increase in all-cause mortality rate in diabetic patients (16), while weight gain after diabetes diagnosis caused a higher risk of CVD (17), chronic kidney disease (CKD) (10), microvascular disease (6), stroke (18) and mortality (19). It is implied from two studies that severe weight fluctuation increases the mortality rate and the risk of microvascular disease (11, 19). Unlike other studies, one study showed that more than 5% weight gain positively affected CVD and coronary heart disease in T2DM patients (14). A summary of studies on the effect of weight change on microvascular and macrovascular complications in patients with T2DM is presented in Table 2.

## Discussion

This systematic review contained articles based on design, duration, type of population (sex-age-race) and herbal preparation, which showed marginally debatable results on the effects of weight change and the risk of microvascular and macrovascular complications in diabetic patients. It should be noted that several articles studied the effects of both weight loss and weight gain, while some only considered weight loss (21, 22) or weight gain (13, 18, 23) as a variable factor to study the risk of microvascular and macrovascular disease. Most of the articles proposed that the role of weight loss in affecting complications is more significant than the effect of weight gain in most patients with T2DM. Xing et al. (24) showed a high risk of all-cause death for weight change among diabetic patients, with weight loss carrying the higher risk. On the contrary, Casanova et al. (12) reported that weight gain and increase in BMI showed worse outcomes than weight loss with respect to microvascular problems in diabetic patients. With this conflict already in place, several studies attempted to determine the underlying factors (25, 26, 28, 32, 33). Williamson et al. (26) showed that the T2DM patients who underwent intentional weight loss had lower total mortality than those with decreased BMI but did not report intentional weight loss.

Similarly, Strelitz et al. (20) claimed that increased physical activity among those who moderately lost weight might have contributed to a lower risk of CVD through inflammatory mechanisms. Intentional weight loss in the early phases of diabetes was also found to reduce the long-term incidence of CVD when applied with intensive glucose control (27). In the case of unintentional weight loss, Murphy et al. (28) suggested that the loss could be due to deteriorating conditions such as sarcopenia, cancer and age-related changes in metabolic function, which could lead to increased mortality. Furthermore, weight loss could reflect lipolysis and impaired insulin action (29). Moreover, Cui et al. (18) showed that the risk factors in the diabetic population were affected by gender and aging. Therefore, the difference between metabolic and health consequences of weight change must be evaluated with respect to gender and age diversity. Based on the study of Drøyvold et al. (30), although weight loss was related to increased mortality in all categories of initial BMI. However, according to sex, the highest mortality rates associated with weight loss were found in men with average weight and overweight women.

When high blood sugar is resistant to medications and other therapies for T2DM, physicians often initiate insulin therapy. This change occurs in about 25% of T2DM patients (31) and weight gain ensues due to the effects of insulin. The increase in BMI is hazardous for blood pressure control, glucose level, lipid profile and inflammatory position. Weight gain has been reported to increase systolic and diastolic blood pressure, triglycerides and oxidative stress, and decrease high-density lipoprotein cholesterol (33). However, the benefits of insulin therapy outweigh the complications related to weight gain in these patients. This could also be attributed to the ‘obesity paradox,’ which describes a negative link between BMI and mortality, and the study examined the effect of weight change on the risk of CVD incidents in patients with T2DM. This study demonstrated that weight gain > 5% is associated with fewer cardiovascular events. This outcome was more significant in adults older than 60 years old. Similarly, Li et al. (33). They reported an inverse relationship between weight gain and stroke risk among the diabetic population. Contrary to previous studies, Kim et al. (32), weight gain greater than 10% increases the risk of macrovascular complications such as stroke. However, this association was only significant in men. Furthermore, the increase in BMI was found to exacerbate kidney damage in diabetic patients, leading to CKD. According to Chung et al. (10). Elevated IL-6 levels in obese patients could interfere with normal kidney function, supporting the results of their study, as weight gain of more than 10% and waist circumference increase of more than 15% were found to be two predictors of developing CKD. Overall, results on this subject vary and some might contradict each other. However, weight loss was a more significant indirect cause of mortality in most. To better understand, future studies are warranted to explore the influence of age, gender and accompanying factors on the link between weight change and diabetes complications.

To our knowledge, this study is the first systematic review to collect data from current articles on the association between weight change and the risk of microvascular and macrovascular complications of diabetes. The strengths of the systematic review are detailed as follows: First, the finding that weight gain is related to vascular complications of diabetes was based on adjusted multivariate data, indicating that the possible association could be drawn independently of potential confounders, such as age, sex, smoking, obesity and concurrent disorders that include hypertension and dyslipidemia. Second, the similarity or difference among the results of the selected studies was not affected by the definition of the terms used. Third, leaving out one study at a time did not significantly affect the results, showing the stability of the findings. Our study also assessed the quality of included articles through the Ottawa New Castle Cheklist and is presented in Table 3.

Our study has limitations that must be taken into account when the results are interpreted. First, considerable heterogeneity was found among the included papers. The potential impact of study methodologies, such as design and setting (based on clinical or population data), and differences in the time intervals in which weight change is measured may contribute to this heterogeneity. Also, although articles with adjusted data were included, the presence of other residual factors is probable and can confuse the outcome.

## Conclusion

In summary, it can be concluded from our analysis that the weight change in patients with T2DM is a valuable predictor of cardiovascular complications. We report that the risk of cardiovascular complications in patients with weight loss is seen to be higher than in patients with weight gain. However, in regular BMI patients, maintaining a stable weight in a healthy range with regular exercise and a healthy diet has the best effect on reducing the risk of CVD. Furthermore, acknowledging the factors that could contribute to the impact of weight change in patients, such as age and gender, could be an essential aspect of our approach to the relationship between weight change and diabetes vascular complications.

## Figures and Tables

**Table 1:** Search strategies for PubMed, Scopus and Google Scholar

PubMed	((BMI[Title/Abstract]) OR (weight[Title/Abstract])) AND ((change[Title/Abstract]) OR (changed[Title/Abstract]) OR (alteration[Title/Abstract]) OR (altered[Title/Abstract]) OR (loss[Title/Abstract]) OR (gain[Title/Abstract]) OR (reduce[Title/Abstract]) OR (reduction[Title/Abstract]) OR (decrease[Title/Abstract]) OR (increase[Title/Abstract])) AND ((Diabetes Mellitus[Title/Abstract]) OR (Diabetes Insipidus[Title/Abstract]) OR (Diabetes[Title/Abstract])) AND ((Microvascular[Title/Abstract]) OR (Macrovascular[Title/Abstract]) OR (Retinopathy[Title/Abstract]) OR (Neuropathy[Title/Abstract]) OR (Foot Ulcer[Title/Abstract]) OR (Stroke[Title/Abstract]) OR (Cardiovascular[Title/Abstract]) OR (Heart[Title/Abstract]))	13,466 articles
Scopus	((TITLE-ABS-KEY ( weight ) OR TITLE-ABS-KEY ( BMI ))) AND ((TITLE-ABS-KEY(change) OR TITLE-ABS-KEY(changed) OR TITLE-ABS-KEY(alteration) OR TITLE-ABS-KEY(altered) OR TITLE-ABS-KEY(loss) OR TITLE-ABS-KEY(gain) OR TITLE-ABS-KEY(decrease) OR TITLE-ABS-KEY(reduce) OR TITLE-ABS-KEY(reduction) OR TITLE-ABS-KEY(increase))) AND ((TITLE-ABS-KEY (microvascular ) OR TITLE-ABS-KEY (macrovascular ) OR TITLE-ABS-KEY (retinopathy ) OR TITLE-ABS-KEY ( neuropathy ) OR TITLE-ABS-KEY (“foot ulcer”) OR TITLE-ABS-KEY (stroke ) OR TITLE-ABS-KEY (Heart))) AND ((TITLE-ABS-KEY (Diabetes Mellitus) OR TITLE-ABS-KEY (Diabetes Insipidus) OR TITLE-ABS-KEY (Diabetes)))	938 articles
Google Scholar	With all of the words: weight, diabetes, change With at least one of the words: microvascular, macrovascular, heart, cardiovascular, retinopathy, neuropathy, nephropathy, ‘foot ulcer’, stroke With all of the words: BMI, diabetes, change With at least one of the words: microvascular, macrovascular, heart, cardiovascular, retinopathy, neuropathy, nephropathy, ‘foot ulcer’, stroke With all of the words: diabetes, change With the exact phrase: ‘body mass index’ With at least one of the words: microvascular, macrovascular, heart, cardiovascular, retinopathy, neuropathy, nephropathy, ‘foot ulcer’, stroke	10,115 articles

**Table 2:** Summary of the studies on the effect of weight change in micro and macro vascular complications in T2DM patients

First author	Year	Type of study	Follow-up duration	Participants: T2DM patients	Gender	Mean age (years old)	Weight change/definition	Weight change assessment method	Outcomes	Adjusted variables	Quality score	Ref.
Chaturvedi	1995	Cohort study	13 years	2,960	52% female	47 ± 2	Gain > 2 kg/m^3^ Loss > 2 kg/m^3^	Measured	Weight loss at BMI < 26 kg/m^2^: mortality rate (RR: 3.05; 95% CI: 1.26, 7.36) Weight loss at BMI > 29 kg/m^2^: mortality rate (RR: 0.84; 95% CI: 0.40, 1.74)	Age-blood pressure-cholesterol-duration of diabetes-retinopathy-smoking status-blood glucose-insulin therapy	7	16
Cho	2002	Cohort study	20 years	5,897	100% female	55 ± 1	Weight loss ≥ 5 Loss = 4.9-Gain = 4.9 Weight gain = 5–7.9 Weight gain = 8–10.9 Weight gain = 11–19.9 Weight gain = 20–29.9 Weight gain = 30–39.9 Weight gain ≥ 40	Self-reported and measured	CHD incidence risk increased as follows: Weight loss ≥ 5: RR≃1.4 Loss = 4.9-Gain = 4.9: RR≃1 Weight gain = 5–7.9: RR≃1.4 Weight gain = 8–10.9: RR≃1.6 Weight gain = 11–19.9: RR≃1.5 Weight gain = 20–29.9: RR≃1.8 Weight gain = 30–39.9: RR≃2 Weight gain ≥ 40: RR≃2.5	Smoking status-age-weight-height-history of myocardial infarction, reproductive history-parental history of myocardial infarction, reproductive history, use of oral contraceptives or postmenopausal hormones, and personal history of coronary heart disease stroke, hypertension, diabetes, elevated serum cholesterol level and cancer	8	17
Chung	2017	Cohort study	7 years	881	52% female	55 ± 9	Weight gain > 10% Weight gain > 5% Stable (5%) Weight loss > 5% Weight loss > 10%	Measured	CKD incidence risk increased as follows: Weight gain > 10%: RR: 1.45; 95% CI: 1.07, 1.97; P = 0.016 Gain > 5% RR: 1.08; 95% CI: 0.79, 1.46; P = 0.634 Loss > 5% RR: 1.08; 95% CI: 0.86, 1.36; P = 0.519 Loss > 10%: RR: 0.91; 95% CI: 0.57, 1.47; P = 0.710	Diabetes duration, gender, education (≤ 6, > 6 years), smoking status (never, past, current smoker) and drinking habit (yes, no), HbA1c, triglycerides and hypertension (high blood pressure or antihypertensive medication use), diabetic retinopathy and albuminuria	7	10
Strelitz	2021	Cohort study	5 years	3,057	42% female	60.2	i) Gained weight > 5% ii) Gained weight > 2%–≤ 5% iii) Maintained weight (gained ≤ 2% or lost < 2%) iv) Lost weight ≥ 2%–< 5% v) Lost weight 5%– < 10% vi) Lost weight ≥ 10%	vi	CVD hazard ratio increased in groups accordingly: i): 0.92 (0.50, 1.70) ii): 1.35 (0.81, 2.24) iii): 1.0 iv): 1.44 (0.87, 2.39) v): 1.05 (0.62, 1.80) vi): 1.50 (0.85, 2.66) All-cause mortality HR increased in groups accordingly: i): 1.27 (0.72, 2.22) ii): 1.31 (0.79, 2.20) iii): 1.0 iv): 1.12 (0.66, 1.92) v): 0.85 (0.47, 1.54) vi): 2.04 (1.17, 3.55) Patients with weight loss of ≥ 10% show significant more all-cause mortality rate	Age, gender, baseline weight, education, smoking, trial group, study center, baseline antihypertensive or lipid-lowering medication use, changes in medication use between baseline and 5 years, and having a CVD event within 5 years after diabetes diagnosis	9 (Out of 11)	19
Moazzeni	2021	Cohort study	14.4 years	763	60.7% female	53.6 ± 11	Lost > 5% weight; lost 3%–5% weight; stable (± 3%) weight; gained 3%–5% weight; gained > 5% weight	Measured	CVD incidence hazard ratios are as followed: Lost > 5% (11%), 3%–5% (11% decrease), gained 3%–5%: (24% decrease), > 5% (30% decrease): not significant; HR and 95% CI, respectively: 1.11 (0.79, 1.56); 0.89 (0.60, 1.33); 0.76 (0.46, 1.23); 0.70 (0.48, 1.01) Age, year: 5% increase BMI: no change (not significant) Current smoker, yes: 64% increase GLD use, yes: 62% increase Family history of premature CVD, yes: 15% increase (not significant) Hypertension, yes: 73% increase Hypercholesterolemia, yes: 77% increase CKD, yes: 22% decrease (not significant) FPG: $4 increase (not significant) HR and 95% CI, respectively; 1.05 (1.03, 1.07); 1.00 (0.97, 1.03); 1.64 (1.03, 2.61); 1.62 (1.21, 2.16); 1.15 (0.84, 1.57); 1.73 (1.32, 2.26); 1.77 (1.27, 2.48); 0.78 (0.58,1.05); 1.04 (1.00,1.08)	Age, sex, body mass index, educational level, current smoking (at first follow-up), glucose-lowering drug use (at baseline or first follow-up), family history of CVD, hypertension, hypercholesterolemia, chronic kidney disease, FPG	9 (Out of 11)	14
Casanova	2020	Cohort study	3.14±0.21 years	154	72.3% female	67.9 (66.6, 69.2)	Weight loss: ≥ 5% Weight gain: ≥ 5%	Measured	Weight loss: 1.2 (95% CI: 13.2, 15.7) AU × min Stable weight: 15.8 (−10.5, −21.0) AU × min Weight gain: 37.8 (−19.4, −56.2) AU × min	Age/sex/SD change	9 (Out of 11)	12
Strelitz	2019	Cohort study	10 years	725	38.5% female	61.1 ± 7.1	i) Gained > 2% weight ii) Maintained weight iii) Lost ≥ 2%–< 5% iv) Lost ≥ 5% weight	Measured	CVD incidence HR: i): 0.41 (0.15, 1.11) ii): 1.00 iii): 0.79 (0.43, 1.46) iv): 0.52 (0.32, 0.86) All-cause mortality HR: i): 1.63 (0.83, 3.19) ii): 1.00 iii): 1.08 (0.60, 1.93) iv): 1.12 (0.52, 2.37) Patients who gained weight were at more risk of all-cause mortality	Age, sex, baseline SES, baseline BMI, smoking at 1 year, use of antihypertensive, lipid- or glucose-lowering medication at 1 year, and trial arm	10 (Out of 11)	15
Polemiti	2021	Cohort study	10.8 years	1,083	46% female	59.1 (52.2–64.4)	i): > 1% BMI loss ii): Stable BMI iii): > 1% BMI gain	Self-reported	HRs and 95% CIs for microvascular and macrovascular complications. Total vascular complications: i): 0.69 (0.54, 0.89) ii): 1.00 (Ref.) iii): 0.86 (0.65, 1.14) Macrovascular complications: i): 1.04 (0.62, 1.74) ii): 1.00 (Ref.) iii): 0.82 (0.42, 1.63) Microvascular complications: i): 0.62 (0.47, 0.80) ii): 1.00 (Ref.) iii): 0.90 (0.67, 1.21) Kidney disease: i): 0.57 (0.40, 0.81) ii): 1.00 (Ref.) iii): 1.03 (0.71, 1.50) Neuropathy: i): 0.73 (0.52, 1.03) ii): 1.00 (Ref.) iii): 0.82 (0.56, 1.20) Patients with increased BMI were at more risk of CVD compared to those with decreased BMI.	Adjusted for age, sex and pre-diagnosis BMI education, smoking status change, smoking duration at pre-diagnosis, smoking duration change, physical activity at pre-diagnosis, physical activity change, alcohol consumption at pre-diagnosis, alcohol consumption change, MedPyr score, lipid-lowering medication, antihypertensive medication and glucose-lowering medication	9 (Out of 11)	6
Cui	2021	Cohort study	7 years	1,774	55.1% female	60.32 ± 8.88	One-unit increase in the BMI level	Measured	OR and P-value for one-unit increase in BMI in different populations for stroke are as followed: Whole population: 1.133 (1.046, 1.242); 0.004 Men: 1.153 (1.045, 1.313); 0.008 Women: 1.12 (0.977, 1.292); 0.12 Increase in BMI was slightly associated with stroke in both sexes	Age, sex (if not stratified), education level, marriage status, residence, exercise, smoking status and drinking hypertension, dyslipidemia, systolic blood pressure, fasting blood glucose, glycosylated haemoglobin, triglycerides, total cholesterol, high-density lipoprotein cholesterol, low-density lipoprotein cholesterol, uric acid and eGFR.	9 (Out of 11)	18
Aucott	2016	Cohort study	5.2 years	2,9316	45.5% female	58 ± 12	i) Loss: 10% or more, < 10%–5%, < 5%–2.5%; ii) Stable: Loss of 2.5% up to gain of 2.5%; iii) Gain: > 2.5%–5%, > 5%–10%, 10% or more.	Measured	HRs for all-cause mortality and cardiovascular outcomes for each weight category: All-cause mortality: i) 1 ii) 0.86 (0.55, 1.33) iii) 0.98 (0.69, 1.37) MI: i) 1 ii) 0.98 (0.62, 1.54) iii) 0.94 (0.64, 1.39) CHF: i) 1 ii) 0.97 (0.54, 1.77) iii) 0.96 (0.59, 1.55) PVD: i) 1 ii) 1.61 (0.87, 2.98) iii) 0.81 (0.43, 1.55) Weight gain and weight loss showed strong association with various kinds of cardiovascular complications.	Age, BMI, sex, smoking status and deprivation	9 (Out of 11)	11
Liu	2020	Cohort study	38 years	173,229	70% female	62.01	Weight gain: < 0 kg 0.1 kg–5 kg > 5 kg	self-reported	Among all recent quitters: HR: 0.83 (95% CI: 0.70, 0.99) Among recent quitters without weight gain: HR: 0.77 (95% CI: 0.62, 0.95) Among recent quitters with weight gain of 0·1 kg–5.0 kg: HR: 0·99 (95% CI: 0.70, 1.41) Among recent quitters with weight gain of > 5·0 kg: HR:0·89 (95% CI: 0·65, 1·23) Among longer-term quitters: HR:0·72 (95% CI: 0.61, 0·84) Among long-term quitters without weight gain: HR: 0.69 (95% CI: 0.58, 0.82) Among long-term quitters with weight gain of 0.1 kg–5.0 kg: HR: 0.57 (95% CI: 0.45, 0.71) Among long-term quitters with weight gain of > 5.0 kg: HR: 0.51 (95% CI: 0.42, 0.62)	Age, diabetes duration, sex, white ethnic origin, BMI assessed in the cycle before diabetes was diagnosed, physical activity, alcohol consumption, Alternative Health Eating Index score, family history of myocardial infarction before age 60 years old, family history of cancer, current aspirin use, current multivitamin use, presence of hypertension, presence of hypercholesterolemia and use of diabetes medication (insulin, oral medication or others)	9 (Out of 11)	13

Note: CVD = cardiovascular disease; HR = hazard ratio; CI = confidence interval; GLD = glucose-lowering drug; BMI = body mass index; RAS = renin-angiotensin system; SBP = systolic blood pressure; CKD = chronic kidney disease; BW = body weight; FPG = fasting plasma glucose; T2DM = type 2 diabetes mellitus; HbA1c = haemoglobin A1c; eGFR = estimated glomerular filtration rate; ACR = albumin to creatinine ratio; CABG = coronary artery bypass graft; PCI = percutaneous coronary intervention; MI = myocardial infarction; LDL = low-density lipoprotein; aHR = adjusted hazard ratio; SD = standard deviation; CHF = congestive heart failure; TM = total mortality; MVE = microvascular events defined as nephropathy, neuropathy or retinopathy; OR = observed ratio; MI = myocardial infarction; PVD = peripheral vascular disease

**Table 3:** Newcastle-Ottawa Scale; all the 11 studies were prospective ones

Authors & year of publication	Selection				Comparability	Outcome			Total score

	a	b	c	d	e	f	g	h	
Chaturvedi & 1995	*	*	*	*	**	* *	*	*	10
Cho & 2002	/	*	/	*	**	*	*	*	7
Chung & 2017	*	*	*	*	**	**	*	*	10
Strelitz & 2021	*	*	*	*	**	**	*	*	10
Moazzeni &2021	*	*	*	*	**	*	*	*	9
Casanova & 2020	*	*	*	*	*	*	*	*	8
Strelitz & 2019	*	*	*	*	**	**	*	*	10
Polemiti & 2021	*	*	/	*	**	*	*	*	8
Cui & 2021	*	*	*	*	*	**	*	*	9
Aucott & 2016	*	*	*	*	**	**	*	*	10
Liu & 2020	/	*	/	*	*	**	*	*	7

Notes: a = representativeness of exposed cohort; b = selection of the non-exposed cohort; c = ascertainment of cohort; d = that outcome of interest not present at the start of the study; e = comparability; f = assessment of outcome; g = follow-upped long enough until the outcomes occur; h = adequacy of follow-up of cohort

## References

[1] Sun B, Luo Z, Zhou J (2021). Comprehensive elaboration of glycemic variability in diabetic macrovascular and microvascular complications. Cardiovasc Diabetol.

[2] Hammes HP (2018). Diabetic retinopathy: hyperglycaemia, oxidative stress and beyond. Diabetologia.

[3] Zhang X, Zhia L, Hambly B, Bao S, Wang K (2017). Diabetic retinopathy: reversibility of epigenetic modifications and new therapeutic targets. Cell Biosci.

[4] Satriano J, Sharma K (2013). Autophagy and metabolic changes in obesity-related chronic kidney disease. Nephrology Dialysis Transplantation.

[5] Kubota T, Kubota N, Kadowaki T (2017). Imbalanced insulin actions in obesity and type 2 diabetes: key mouse models of insulin signaling pathway. Cell Met.

[6] Polemiti E, Baudry J, Kuxhaus O, Jäger S, Bergmann MM, Weikert C (2021). BMI and BMI change following incident type 2 diabetes and risk of microvascular and macrovascular complications: the EPIC-Potsdam study. Diabetologia.

[7] Rossi MC, Nicolucci A, Pellegrini F, Comaschi M, Ceriello A, Cucinotta D (2010). Obesity and changes in urine albumin/creatinine ratio in patients with type 2 diabetes: the DEMAND study. Nutr Metabol Cardiovascular Dis.

[8] Svensson MK, Tyrberg M, Nyström L, Arnqvist HJ, Bolinder J, Östman J (2015). The risk for diabetic nephropathy is low in young adults in a 17-year follow-up from the Diabetes Incidence Study in Sweden (DISS). Older age and higher BMI at diabetes onset can be important risk factors. Diabetes Metab Res Rev.

[9] Huang WH, Chen CY, Lin JL, Lin-Tan DT, Hsu CW, Yen TH (2014). High body mass index reduces glomerular filtration rate decline in type II diabetes mellitus patients with stage 3 or 4 chronic kidney disease. Medicine.

[10] Chung HF, Al Manun A, Huang MC, Long KZ, Huang YF, Shin SJ (2017). Obesity, weight change, and chronic kidney disease in patients with type 2 diabetes mellitus: a longitudinal study in Taiwan. J Diabetes.

[11] Aucott LS, Philip S, Avenell A, Afolabi E, Sattar N, Wild S (2016). Patterns of weight change after the diagnosis of type 2 diabetes in Scotland and their relationship with glycaemic control, mortality and cardiovascular outcomes: a retrospective cohort study. BMJ Open.

[12] Casanova F, Gooding KM, Shore AC, Adingupu DD, Mawson D, Ball C (2020). Weight change and sulfonylurea therapy are related to 3 year change in microvascular function in people with type 2 diabetes. Diabetologia.

[13] Liu G, Hu Y, Zong G, Pan A, Manson JE, Rexrode KM (2020). Smoking cessation and weight change in relation to cardiovascular disease incidence and mortality in people with type 2 diabetes: a population-based cohort study. Lancet Diabetes Endocrinol.

[14] Moazzeni SS, Arani RH, Deravi N, Hasheminia M, Khalili D, Azizi F (2021). Weight change and risk of cardiovascular disease among adults with type 2 diabetes: more than 14 years of follow-up in the Tehran Lipid and Glucose Study. Cardiovasc Diabetol.

[15] Strelitz J, Ahern AL, Long GH, Hare MJL, Irving G, Boothby CE (2019). Moderate weight change following diabetes diagnosis and 10 year incidence of cardiovascular disease and mortality. Diabetologia.

[16] Chaturvedi N, Fuller JH, The WHO Multinational Study Group (1995). Mortality risk by body weight and weight change in people with NIDDM. The WHO multinational study of vascular disease in diabetes. Diabetes Care.

[17] Cho E, Manson JE, Stampfer MJ, Solomon CG, Colditz GA, Speizer FE (2002). A prospective study of obesity and risk of coronary heart disease among diabetic women. Diabetes Care.

[18] Cui C, Wu Z, Shi Y, Xu Z, Zhoa B, Zhou D (2021). Sex-specific association of BMI change with stroke in middle-aged and older adults with type 2 diabetes. Nutr Metab Cardiovasc Dis.

[19] Strelitz J, Sharp SJ, Khunti K, Vos RC, Rutten GEH, Webb DR (2021). Association of weight loss and weight loss maintenance following diabetes diagnosis by screening and incidence of cardiovascular disease and all-cause mortality: An observational analysis of the ADDITION-Europe trial. Diabetes Obes Metab.

[20] Strelitz J, Ahern AL, Long GH, Hare MJL, Irving G, Boothby CE (2019). Moderate weight change following diabetes diagnosis and 10 year incidence of cardiovascular disease and mortality. Diabetologia.

[21] Doehner W, Erdmann E, Cairns R, Clark AL, Dormandy JA, Ferrannini E (2012). Inverse relation of body weight and weight change with mortality and morbidity in patients with type 2 diabetes and cardiovascular co-morbidity: an analysis of the PROactive study population. Int J Cardiol.

[22] Chan YH, Chen SW, Chao TF, Kao YW, Huang CY, Chu PH (2021). The risk of consequent nephropathy following initial weight loss in diabetic patients treated with sodium glucose cotransporter 2 inhibitors. Cardiovasc Diabetol.

[23] de Mutsert R, Sun Q, Willet WC, Hu FB, van Dam RM (2014). Overweight in early adulthood, adult weight change, and risk of type 2 diabetes, cardiovascular diseases, and certain cancers in men: a cohort study. Am Journal Epidemiol.

[24] Xing Z, Pei J, Huang J, Peng X, Chen P, Hu X (2019). Weight change is associated with increased all-cause mortality and non-cardiac mortality among patients with type 2 diabetes mellitus. Endocrine.

[25] Chiriboga DE, Ma Y, Li W, Olendzki BC, Pagoto SL, Merriam PA (2008). Gender differences in predictors of body weight and body weight change in healthy adults. Obesity (Silver Spring).

[26] Williamson DF, Thompson TJ, Thun M, Flanders D, Pamuk E, Byers T (2000). Intentional weight loss and mortality among overweight individuals with diabetes. Diabetes Care.

[27] Holman RR, Paul SK, Bethel MA, Matthews DR, Neil HAW (2008). 10-year follow-up of intensive glucose control in type 2 diabetes. N Eng J Med.

[28] Murphy RA, Patel KV, Kritchevsky SB, Houston DK, Newman AB, Koster A (2014). Weight change, body composition, and risk of mobility disability and mortality in older adults: a population-based cohort study. J Am Geriatr Soc.

[29] Luk AOY, So WY, Ma RCW, Kong APS, Ozaki R, Ng VSW (2008). Metabolic syndrome predicts new onset of chronic kidney disease in 5,829 patients with type 2 diabetes: a 5-year prospective analysis of the Hong Kong Diabetes Registry. Diabetes Care.

[30] Drøyvold WB, Lund Nilsen TI, Lydersen S, Midthjell K, Nilsson PM, Nilsson JÅ (2005). Weight change and mortality: the Nord-Trøndelag Health Study. J Intern Med.

[31] Hodish I (2018). Insulin therapy, weight gain and prognosis. J Pharmacol Ther.

[32] Kim MK, Han K, Koh ES, Kim ES, Lee MK, Nam GE (2019). Weight change and mortality and cardiovascular outcomes in patients with new-onset diabetes mellitus: a nationwide cohort study. Cardiovasc Diabetol.

[33] Li W, Katzmarzyk PT, Horswell R, Zhang Y, Zhao W, Wang Y (2015). Body mass index and stroke risk among patients with type 2 diabetes mellitus. Stroke.

